# A cohort study of mild encephalitis/encephalopathy with a reversible splenial lesion in children

**DOI:** 10.1002/brb3.2306

**Published:** 2021-08-01

**Authors:** Jiao Xue, Ying Zhang, Jie Kang, Chongfeng Duan, Zhi Yi, Chengqing Yang, Fei Li, Kaixuan Liu, Zhenfeng Song

**Affiliations:** ^1^ Department of Pediatric Neurology and Endocrinology the Affiliated Hospital of Qingdao University Qingdao Shandong China; ^2^ Department of Pediatric Emergency the Affiliated Hospital of Qingdao University Qingdao Shandong China; ^3^ Department of Radiology the Affiliated Hospital of Qingdao University Qingdao Shandong China

**Keywords:** child, magnetic resonance imaging, mild encephalitis/encephalopathy with a reversible splenial lesion (MRES)

## Abstract

**Purpose:**

To investigate the clinical features, imaging features, and prognosis of mild encephalitis/encephalopathy with a reversible splenial lesion (MERS) in children

**Methods:**

The clinical and imaging data of a cohort of 28 children diagnosed as MERS from January 2019 to October 2020 were retrospectively analyzed

**Results:**

Of the 28 patients, 17 were males and 11 were females. The onset age ranged from 8 months to 12 years old, with an average age of 4 years and 2 months. All children developed normally before onset, and three of them had a history of febrile convulsion. More than half of the patients (62.9%) had preceding infections of gastrointestinal tract. All the cases developed seizures, and most (71.4%) had more than one time. Other neurological symptoms included dizziness/headache, consciousness disorder, limb weakness, blurred vision, and dysarthria. Cranial magnetic resonance imaging (MRI) showed lesions in the splenium of the corpus callosum in all, extending to other areas of the corpus callosum, bilateral semi‐ovoid center, and adjacent periventricular in two cases. The clinical symptoms were relieved after steroids, intravenous immunogloblin, and symptomatic treatment, without abnormal neurodevelopment during the followed‐up (2 months–2 years). Complete resolution of the lesions was observed 8–60 days after the initial MRI examinations

**Conclusion:**

MERS in children is related to prodromal infection mostly, with a wide spectrum of neurologic symptoms, characteristic MRI manifestations, and good prognosis.

## Graphical abstract

We retrospectively analyzed the features of a cohort of 28 children with MERS, and summarized a total of 183 patients reported in the past 5 years. MERS in children is related to prodromal infection in most, presented with a wide spectrum of neurologic symptoms, characteristic MRI manifestations and good prognosis.

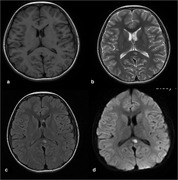

## 1 INTRODUCTION

Mild encephalitis/encephalopathy with a reversible splenial lesion (MERS) is a clinico‐radiological syndrome first described by Tada et al. (2004) in 2004, which typically present with mild encephalopathy following prodromal symptoms, such as fever, cough, vomiting, and/or diarrhea. The most pronounced neurologic symptoms are disturbance of consciousness, abnormal speech, delirious behavior, seizures, muscle weakness, ophthalmoplegia, facial nerve paralysis, and headache (Ueda et al., 2016; Fang et al., 2017), which usually recover within a month (Takanashi, 2009). Magnetic resonance imaging (MRI) is characterized by a reversible lesion with homogenously reduced diffusion in the corpus callosum (at least involving the splenium), sometimes associated with symmetrical white matter lesions (Takanashi, 2009). Here, we retrospectively analyzed the clinical and imaging data of 28 children diagnosed as MERS, and discussed the clinico‐radiological features and prognosis.

## 2 PATIENTS AND METHODS

After searching the inpatient medical record system of our pediatrics department (admitting patients under 18 years old) between January 2019 and October 2020, 28 children diagnosed as MERS were retrospectively collected. The diagnosis of MERS was based on the following criteria (Hoshino et al., 2012): (1) onset with neuropsychiatric symptoms, such as abnormal speech and/or behavior, impaired consciousness, and convulsion, usually within 1 week after the onset of fever, cough, vomiting and/or diarrhea, and so on; (2) complete recovery without sequelae, mostly within 10 days after the onset of neuropsychiatric symptoms; (3) high signal‐intensity lesion in the splenium of corpus callosum, in the acute stage. T1 and T2 signal changes are mild; (4) lesion may involve the entire corpus callosum and the cerebral white matter in a symmetric fashion; and (5) lesion disappears with neither residual signal changes nor atrophy.

The data, including clinical manifestations, treatment processes, and outcomes, were collected. Complete blood count, electrolytes, ammonia, lactic acid, C‐reactive protein, and liver and renal function examinations were performed. Hyponatremia is defined as a serum Na^+^ less than 135 mmol/L. Among them, twenty‐five patients underwent lumbar puncture, and routine, biochemical, smear test, and bacterial culture of cerebrospinal fluid (CSF) were examined. Cranial MRI was performed and rechecked in all. Video electroencephalography (EEG) was performed in 23 patients. Follow‐up was conducted through telephone or return visit for a period of 2–24 months. The psychomotor development was assessed according to clinical judgment. In addition, we browsed the published papers by using “MERS,” “corpus callosum,” “reversible splenial lesion” as keywords in Wanfang, CNKI database, from January 2016 to December 2020. All obtained articles were reviewed to identify that the cases met the diagnostic criteria of MERS. Eventually, a total of 183 children cases with relatively comprehensive clinico‐radiological data were retrieved.

## 3 RESULTS

### 3.1 General information

Of the 28 patients, seventeen were males and 11 were females. All children developed normally previously, and three of them had a history of febrile convulsion. The onset age ranged from 8 months to 12 years old, with an average age of 4 years and 2 months. Twenty‐four cases developed in winter and spring, four in summer and autumn.

### 3.2 Clinical manifestation

More than half of the patients (18/28 cases, 62.9%) had preceding infections of gastrointestinal tract, presenting as fever, vomiting, abdominal pain, and diarrhea predominantly. The interval from infection onset to the presentation of nervous system symptoms, such as convulsions, was 1–8 days, with an average of 4 days. The vast majority (24/28 cases, 85.7%) had fever. All the cases developed seizures: 8 cases (28.6%) had only one convulsion; 20 cases (71.4%) had equal to or more than two times of convulsions. Some patients were accompanied by other neurological symptoms, including dizziness/headache (12 cases), consciousness disorder (8 cases), limb weakness (3 cases), blurred vision (2 cases), and dysarthria (1 case) (Table 1).

**TABLE 1:** Summary of clinical manifestations of the 28 MERS cases

Category	Data
Onset age (average age)	8 months–12 years (4 years and 2 months)
Gender (*n*, %)	
Female	11 (39.3)
Male	17 (60.7)
Clinical manifestations (*n*, %)	
Prodromal symptoms	
Fever	24 (85.7)
Diarrhea and abdominal pain	18 (62.9)
Vomiting	15 (53.6)
Seizure	28 (100)
One time	8 (28.6)
≥2 times	20 (71.4)
Dizziness/headache	12 (42.9)
Consciousness disorder	8 (28.6)
Limb weakness	3 (10.7)
Blurred vision	2 (7.1)
Dysarthria	1 (3.6)

### 3.3 Auxiliary examination

Mild hyponatremia was presented in 17 patients (125–134 mmol/L), including 5 cases with 125–129 mmol/L and 12 cases with 130–134 mmol/L. Elevated glutamic‐pyruvic transaminase and glutamic‐oxalacetic transaminase were found in 2 cases. Elevated creatine kinase isoenzyme (35–117 U/L) was found in 6 cases. Only 10 patients had clinically proven pathogen infection, including rotavirus in 4 cases, influenza virus, respiratory syncytial virus, and mycoplasma pneumoniae (MP) in 2 cases, respectively (Table 2).

**TABLE 2:** Summary of auxiliary examination and follow‐up of the 28 MERS cases

Category	Data
Pathogen infection (*n*, %)	10 (35.7)
Rotavirus	4 (14.3)
Influenza virus	2 (7.1)
Respiratory syncytial virus	2 (7.1)
Mycoplasma pneumoniae	2 (7.1)
Hyponatremia (*n*, %)	17 (60.7)
130–134 mmol/L	12 (42.9)
125–129 mmol/L	5 (17.9)
Abnormal liver function (*n*, %)	2 (7.1)
Abnormal myocardial enzyme (*n*, %)	6 (21.4)
Abnormal CSF (*n*, %)	4 (14.3)
Abnormal EEG (*n*, %)	8 (28.6)
Abnormal MRI (*n*, %)	28 (100)
Type I	26 (92.9)
Type II	2 (7.1)
Follow‐up	
Duration	2 months–2 years
Normal neurodevelopment (*n*, %)	28 (100)
Lesion disappearance	8–60 days

Of the 25 patients who received lumbar puncture examinations, only four showed abnormal results, including 3 cases with elevated CSF pressure and one case with increased CSF cell count (white blood cells: 110×10^6^/L, and the monocyte: 100×10^6^/L). The CSF protein, sugar, and chloride were normal, and CSF etiology was negative.

Eight of the 23 patients who underwent video EEG examination showed abnormal results, presenting as medium to high amplitude bilateral paroxysmal slow waves.

**FIGURE 1:**
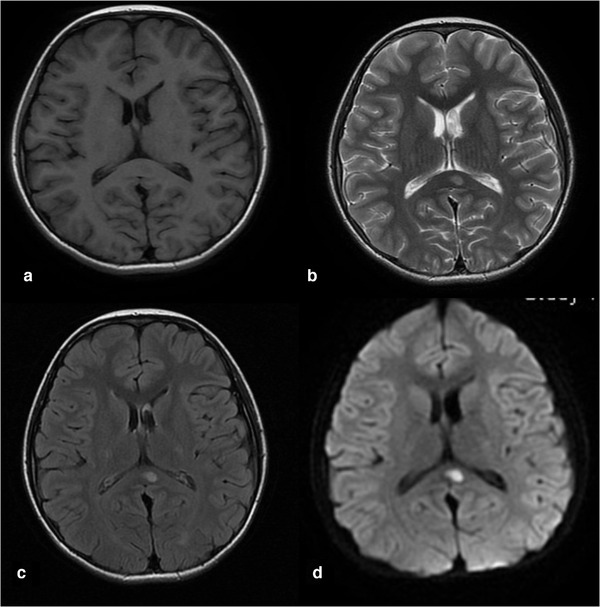
Cranial MRI revealed an isolated elliptic lesion in the splenium of the corpus callosum. The lesions were isointense to slightly hypointense on T1‐weighted images (a), homogeneously hyperintense on T2‐weighted images (b) and DWI (d), slightly hyperintense on T2‐FLARE (c)

**FIGURE 2:**
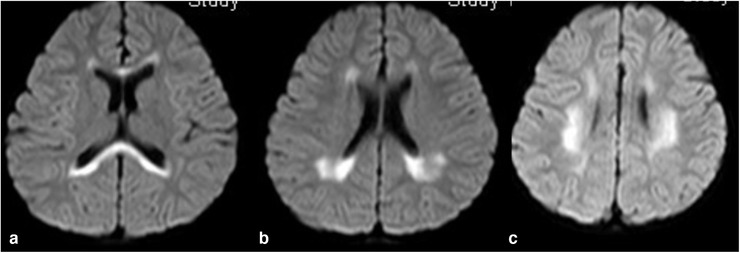
Cranial MRI revealed lesion extending to other areas of the corpus callosum (a), adjacent peri ventricular (b), and bilateral semi‐ovoid center (c)

All the patients received the first cranial MRI examination at 3–10 days (mean 5.4 days) of the course. Twenty‐six patients revealed an isolated oval lesion in the splenium of corpus callosum (type I, Fig. 1), and 2 patients revealed lesions extending to the whole corpus callosum, bilateral semi‐ovoid center, and adjacent periventricular (type II, Fig. 2). The lesions were homogeneously hyperintense on diffusion‐weighted imaging (DWI) and T2‐weighted images, slightly hyperintense on T2‐FLARE, isointense to slightly hypointense on T1‐weighted images, and showed reduced diffusion without contrast enhancement.

### 3.4 Therapy and prognosis

The clinical symptoms were relieved after symptomatic treatment, such as anti‐infection, reducing cranial pressure and sedation. In addition, twenty‐six patients received intravenous or oral steroid (methylprednisone or prednisone) for 7–10 days, with a total dose of less than 1.5 mg/(kg·d) (converted to prednisone dose). One of them had limb weakness, dysarthria, and increased CSF cell count, and was further treated with 2 g/kg intravenous immunogloblin (IVIG) in 3 days.

Follow‐up period ranged from 2 months to 2 years. All the children showed no abnormal neurodevelopment. There was complete resolution of the lesions in all the patients 8–60 days after the initial examinations.

### 3.5 Literature review

Considering the small number of cases here might not fully represent the characteristics of Chinese patients, we browsed the papers on MERS published in China from January 2016 to December 2020, and a total of 183 children cases were retrieved (166 cases with type I and 17 cases with type II) (Duan et al., 2020; Chen et al., 2020; Yu et al., 2019; Zhang et al., 2015; He and Zhu, 2016; Zhang et al., 2017; Wu et al., 2017; Dong et al., 2017; Hu et al., 2016; Tao et al., 2017; Li et al., 2020; Li and Sheng, 2017; Zhang et al., 2017). Of them, ninty‐nine were males and 84 were females. The onset age ranged from 6 months to 17 years old. The vast majority (112/122 cases, 91.8%) had prodromal infections, including gastrointestinal tract infection (74.1%), respiratory infection (25%), and skin infections (0.9%). And half of them (74/147 cases, 50.3%) had definite pathogen, including rotavirus (47.3%), MP (32.4%), salmonella, coxsackievirus, influenza virus, respiratory syncytial virus, streptococcus, and herpes zoster virus. Convulsions (69%) and headaches (18%) were the most common neurological symptoms. Hyponatremia was found in 33 cases, and abnormal CSF and EEG were found in 24% and 44.6% of the patients, respectively. All the patients had good prognosis and no sequelae (Table 3).

**TABLE 3:** Summary of clinical features of 183 MERS cases published in literatures

Category	Data
Gender (*n*, %)	183
Female	84 (45.9)
Male	99 (54.1)
Prodromal symptoms	112/122 with data available
Gastrointestinal tract	83 (74.1)
Respiratory	28 (25)
Skin	1 (0.8)
Pathogen infection (*n*, %)	74/147 with data available
Rotavirus	35 (47.3)
Mycoplasma pneumoniae	24 (32.4)
Salmonella	5 (6.7)
Coxsackievirus	4 (5.4)
Influenza virus	2 (2.7)
Respiratory syncytial virus	2 (2.7)
Streptococcus	1 (1.4)
Herpes zoster virus	1 (1.4)
Clinical symptoms (*n*)	100 cases with data available
Convulsion	69
Headaches	18
Fever	15
Blurred vision	5
Limbs numb	1
Hyponatremia (*n*)	33
Abnormal liver function (*n*)	8
Abnormal myocardial enzyme (*n*)	20
Abnormal CSF (*n*, %)	31/129 with data available (24)
Abnormal EEG (*n*, %)	37/83 with data available (44.6)
Abnormal MRI (*n*, %)	183 (100)
Type I	166 (90.7)
Type II	17 (9.3)

## 4 DISCUSSION

MERS is a clinico‐radiological entity with varied etiologies, characterized by a reversible lesion with homogeneously reduced diffusion in the corpus callosum, and sometimes associated with symmetrical white matter lesions on neuroimaging (Takanashi, 2009). Here, we retrospectively analyzed the features of a cohort of 28 children with MERS, and summarized a total of 183 Chinese patients reported in the past 5 years (Duan et al., 2020; Chen et al., 2020; Yu et al., 2019; Zhang et al., 2015; He & Zhu, 2016; Zhang et al., 2017; Wu et al., 2017; Dong et al., 2017; Hu et al., 2016; Tao et al., 2017; Li et al., 2020; Li and Sheng, 2017; Zhang et al., 2017), verifying that the clinical characteristics were generally consistent.

The most common cause of MERS in children is infection (Ueda et al., 2016). Various infectious agents have been reported to associate with MERS in children, such as rotavirus, adenovirus, influenza A and B, Epstein–Barr virus, Dengue virus, mumps virus, herpes simplex virus, parainfluenza, parvovirus B‐19, and cytomegalovirus, as well as MP, *Streptococcus pneumoniae*, *Campylobacter jejuni*, and so on (Chen et al., 2016; Karampatsas et al., 2015; Fong et al., 2017; Avcu et al., 2017; Abenhaim Halpern et al., 2013; Suzuki et al., 2014; Yıldız et al., 2018). In our cohort, 35.7% of the patients had proven infectious agents, with viral infection, especially rotavirus (40%, 4/10 cases), being the most common. Similarly, the vast majority of patients reviewed from published papers had gastrointestinal tract or respiratory prodromal infection caused by rotavirus or MP, respectively (Table 3).

Though varied origin is associated with callosal lesions, including infection, high‐altitude cerebral edema, seizures, antiepileptic drug use and withdrawal, and metabolic disturbances (Yıldız et al., 2018). There is no evidence to support any other cause but MERS, combined with the onset, clinical manifestations, imaging features, and prognosis of our cases. The mechanism by which the lesions occur in MERS, especially the selective lesions in the splenium of the corpus callosum, remains elusive. Possible pathogenesis includes intramyelinic edema due to separation of myelin layers, interstitial edema in tightly packed fibers, and a transient inflammatory infiltrate (Tada et al., 2004; Takanashi, 2009). Although 35.7% of our patients were verified with infectious agents, no causative agent was found in CSF cultures, suggesting that it was an indirect mechanism, such as immune‐mediated mechanism (Takanashi, 2009) rather than direct invasion of pathogen that lead to the lesions. And the release of myelin‐specific neurotoxins by the pathogen might cause inflammatory infiltrate and further lead to transient cerebral edema (Yıldız et al., 2018). Hyponatremia is another possible etiologic factor in MERS. The rate of hyponatremic patients in our study (60.7%) was similar to the rate reported previously (Takanashi, 2009). It was postulated that hypotonic hyponatremia might result in entry of water into the brain and lead to intramyelinic edema, causing transient reduced diffusion seen on MRI (Takanashi, 2009).

In our patients, the prodromal symptoms were mainly fever, diarrhea, abdominal pain, and vomiting, with fever as the most common (85.7%), which was generally similar to those reported previously (Ueda et al., 2016; Fang et al., 2017). Consistent with published reports, seizure was the most common presenting symptom (Yıldız et al., 2018). It presented in all our patients and 69% of the patients reviewed from literatures, and occurred more than one time in most patients (71.4%). The other neurologic symptoms here included dizziness/headache, consciousness disorder, limb weakness, blurred vision, dysarthria, and limbs numb. The corpus callosum is the biggest fiber bundle, with projections into prefrontal, premotor, primary motor, and primary sensory areas. The splenium is the posterior part of the corpus callosum, connecting different cortical areas, including occipital, parietal, and temporal lobes (Knyazeva, 2013). The disturbance in the collosal connections can cause disorder of motor control, spatial orientation, vision, hearing, and language‐related behaviors (Jea et al., 2008), which may explain the neurologic symptoms of our patients here, including the limb weakness, blurred vision, and dysarthria.

There is no treatment guideline for MERS by now. Though methylprednisolone pulse therapy and IVIG are recommended for patients with infectious encephalopathy regardless of pathogen or clinicoradiological syndromes (Mizuguchi et al., 2007), their efficacy on MERS is still lacking. In the present study, 26 cases were treated with steroids, one case was treated with IVIG and two cases received symptomatic treatment only. And all the patients here recovered completely without neurological sequelae at the last follow‐up. Previous reports by Yuan et al. (2017) showed that most of MERS patients could completely clinically recover irrespective of treatment, which suggested that methylprednisolone pulse therapy or IVIG treatments might not be necessary. However, the limb weakness and dysarthria in one case here were not completely relieved after steroid treatment, and further remission after IVIG treatment. Therefore, further studies on the role of steroid and IVIG are needed in the future.

In the literature, patients with type I MERS were generally reported to recover completely both clinically and on imaging (Karampatsas et al., 2015; Fong et al., 2017; Avcu et al., 2017; Abenhaim Halpern et al., 2013; Suzuki et al., 2014; Yıldız et al., 2018; Pan et al., 2015). However, type II lesions on MRI might persist for months even if their size diminished, and some patients with type II MERS could develop neurologic sequelae (Yıldız et al., 2018; Pan et al., 2015). The vast majority of our patients had type I lesions, and only two patients had type II lesions. All of our patients recovered completely without any clinical sequelae at the last follow‐up. And the full normalization of MRI was shown at 8 days–2 months.

In conclusion, MERS occurs predominantly in young children and is related to prodromal infection in most. A wide spectrum of neurologic symptoms were presented, including seizure, dizziness/headache, limb weakness, blurred vision, dysarthria, and so on. Characteristic MRI manifestations normalize in days to months with good prognosis.

## CONFLICT OF INTEREST

The authors declare no conflict of interest.

## ETHICAL APPROVAL

The study was approved by the Ethical Committee of Affiliated Hospital of Qingdao University. The children's parents had given written informed consent to publish these case details.
